# Comparative biological properties of resin-free and resin-based calcium silicate-based endodontic repair materials on human periodontal ligament stem cells

**DOI:** 10.1007/s00784-023-05288-5

**Published:** 2023-10-05

**Authors:** Shehabeldin M. Saber, Shaimaa M. Gomaa, Mohamed M. Elashiry, Ahmed El-Banna, Edgar Schäfer

**Affiliations:** 1 Department of Endodontics, Faculty of Dentistry, The British University in Egypt (BUE), Cairo, Egypt; 2 Dental Science Research Group, Health Research Centre of Excellence, The British University in Egypt (BUE), Cairo, Egypt; 3 Department of Endodontics, Faculty of Dentistry, Ain Shams University, Egypt, Cairo, Egypt; 4 Department of Endodontics, Dental College of Georgia, Augusta University, Augusta, Georgia USA; 5 Department of Biomaterials, Faculty of Dentistry, Ain Shams University, Cairo, Egypt; 6 Central Interdisciplinary Ambulance in the School of Dentistry, University of Münster, Münster, Germany

**Keywords:** Calcium ion release, Calcium silicate-based sealers, Cell migration, Cytocompatibility, Periodontal ligament stem cells

## Abstract

**Objectives:**

To investigate the effect of three different calcium silicate-based materials (CSBM) on the biological behavior of human periodontal ligament stem cells (hPDLSCs).

**Methods:**

Eluates of Biodentine, NeoPutty and TheraCal PT prepared at 1:1, 1:2, and 1:4 ratios were extracted under sterile conditions. The cytotoxicity of the extracts to the hPDLSCs was assessed using the MTT assay. Scratch wound healing assay was utilized for assessing cell migration. Scanning electron microscopy was used to detect cell attachment and morphology. Calcium ion release was measured using inductively coupled plasma-optical emission spectrometry; the pH-value was evaluated with a pH-meter. ANOVA with post hoc Tukey test was used for statistical analysis.

**Results:**

Cell viability was significantly higher for Biodentine and NeoPutty at day 1 with all dilutions (*p* < 0.05), while at day 3 and day 7 with dilutions 1:2 and 1:4; all materials showed similar behavior (*p* > 0.05). Biodentine had the highest percentage of cell migration into the scratched area at day 1 for all dilutions (*p* < 0.05). Stem cells were attached favorably on Biodentine and NeoPutty with evident spreading, and intercellular communications; however, this was not shown for TheraCal PT. Biodentine showed the highest pH values and calcium ion release (*p* < 0.05).

**Conclusions:**

The resin-free CSBM showed better performance and favorable biological effects on hPDLSCs and were therefore considered promising for usage as endodontic repair materials.

Clinical significance: Proper selection of materials with favorable impact on the host stem cells is crucial to ensure outcome in different clinical scenarios.

## Introduction

Calcium silicate-based materials (CSBMs) have been implicated as the biomaterial of choice for different endodontic procedures including their use to repair pathologic or iatrogenic perforations [1], as orthograde apical plugs and for retrograde apical sealing [2, 3], as coronal plugs during regenerative endodontic procedures [3] and for vital pulp therapy [4]. During such clinical applications, they come in contact and interact with different host stem cell populations where they should ideally promote a biological response that favors cell survival, proliferation, differentiation [5, 6], and consequently tissue repair or regeneration by osteogenic differentiation of dental stem cells [7–9].

Biodentine (Septodont, Saint-Maur-des-Fossés, France) is a bioactive tricalcium silicate-based material designed as a dentine substitute with special hydration kinetics that improved its mechanical strength, initial and final setting time, and discoloration potential in comparison with Portland-based cements [10]. Biodentine displayed favorable biological interactions with different host cell populations including human dental pulp stem cells (hDPSCs) [11], human bone marrow-derived mesenchymal stem cells (hBMSCs) [12], stem cells from human exfoliated deciduous teeth (SHED) [13], stem cells of the apical papilla (SCAP) [14], periodontal ligament fibroblasts (hPDLFs) [15], and human gingival fibroblasts [16].

NeoPutty (Avalon Biomed, Bradenton, USA) is a recently introduced premixed CSBM consisting of an extremely fine, inorganic powder of tricalcium/dicalcium silicate in a water-free organic liquid designed to set *in vivo* in the presence of moisture from the surrounding apical tissues, dentinal tubules, or pulp. Favorable cytocompatibility of NeoPutty has been reported with host cells including human dental pulp cells (DPCs) [17], hDPSCs [18, 19], and hPDLFs [18].

TheraCal PT (Bisco, Inc., Schamburg, IL, USA) is a new dual-cured, resin modified calcium silicate material designed for vital pulp therapy procedures. It has been shown that TheraCal PT possesses an improved *in vitro* cytocompatibility and mineralization potential on hDPSCs compared with its predecessor TheraCal LC [20]. These findings were corroborated by another study using also hDPSCs [21]. Biological properties of TheraCal PT were found to be comparable to those of Biodentine [20] and MTA [21]. The interaction between TheraCal PT and hPDLSCs is not yet studied, which if found favorable can expand the use of this material in root repair procedures.

This study aimed to evaluate the cytocompatibility of Biodentine, NeoPutty, and TheraCal PT in terms of viability, migration, and attachment of hPDLSCs. Moreover, the three CSBMs were also investigated regarding their pH value and their calcium ion release. The null hypothesis was that there is no difference between the tested materials regarding their cytocompatibility on hPDLSCs, pH value, and calcium ion release.

## Materials and methods

### Material preparation

The tested materials in this study were as follows: Biodentine, NeoPutty, and TheraCal PT (Table 1). Materials were prepared as discs according to the manufacturer’s instructions under sterile conditions. The materials were placed into a custom-made polyvinylsiloxane rubber mould (diameter 10 mm and thickness 1 mm), separately, and left undisturbed to set at 37 °C and 5% CO_2_ and 95 h. Discs were then exposed to ultraviolet light for 30 min on every side for sterilization.

**Table 1:** Composition of materials used in this study

Product	Description	Form of supply	Composition	Manufacturer and lot number
Biodentine	Chemical cured by hydration tri-calcium silicate	Powder capsule + vial of special liquid	Powder: Tricalcium silicate, dicalcium silicate, calcium carbonate, oxide filler, iron oxide shade, and zirconium oxide. Liquid: Water, calcium chloride, and hydrosoluble polymer	Septodont, Saint-Maur-des-Fosses Cedex, France B27049
NeoPutty	Single paste chemically cured by hydration tricalcium silicate	Premixed putty syringe	Tantalite Tricalcium silicate Dicalcium silicate Calcium aluminate Grossite Tricalcium aluminate Calcium sulfate	Avalon Biomed, Houston, TX, USA 2021120804
TheraCal PT	Dual cured resin modified tri-calcium silicate.	Automixing dual cure syringe	Silicate glass cement, polyethylene glycol dimethacrylate, Bis-GMA, barium zirconate	Bisco, Schaumburg, IL, USA 1900003528

In accordance with the International Organization for Standardization (ISO), the eluates of the different materials were extracted under sterile conditions, using Dulbecco’s modified Eagle’s medium (DMEM; Sigma-Aldrich, St. Louis, MO, USA) as an extraction vehicle. The extraction procedure was performed as follows: the materials were stored in the culture medium for 24 h at 37 °C in a humid atmosphere containing 5% CO_2_ with agitation. The ratio of material surface area to medium volume was set at approximately 3 cm^2^/ml in accordance with ISO10993-12 standards. Finally, serial dilutions of the extraction medium were prepared at 1:1, 1:2, and 1:4 ratios [22].

### Isolation and culture of periodontal ligament (PDL) cells

Human permanent teeth were obtained from healthy donors whose teeth were extracted for orthodontics reason at the Maxillofacial Surgery Department, Faculty of Dentistry, under the approval of the Ethical Committee of Faculty of Dentistry, The British University in Egypt (FD BUE REC 22-005 on 23/1/2022 valid to 23/1/2023) and obtaining informed consent from the patients after clear explanation by the primary investigator regarding the purpose of the study and that it is a volunteering action supporting the field of research instead of wasting the extracted teeth and being incinerated. The patients were informed with the study information privately and the primary investigator confirmed the patient’s data confidentiality.

The extracted teeth (*n* = 10) were sound and surrounded by a healthy periodontium. After extraction, the teeth were stored in DMEM supplemented with antibiotics (300 U/ml penicillin and 300 mg/ml streptomycin; Sigma). Primary cultures were conducted maximum within 24 h after extraction. PDL tissues were separated from the root surface of healthy extracted third molars and sterilized with phosphate-buffered saline (PBS, Sigma-Aldrich) supplemented with antibiotics at decreasingly concentrations.

These PDL tissues were cultured by the outgrowth method, in which small fragments of PDL were placed onto 35-mm dishes with 1 ml complete culture medium prepared from Dulbecco’s Modified Eagle Medium (DMEM)/nutrient mixture F-12 Ham medium (DMEM/F12, Sigma) supplemented with 10% fetal bovine serum (FBS, Gibco, Grand Island, NY, USA). 1% L-glutamine, 100-U/ml penicillin, and 100 μg/ml streptomycin (Gibco). Finally, cells were incubated in a humid environment of 5% CO_2_ at 37 °C. Cell growth and morphology were observed under an inverted microscope (Olympus IX71, Tokyo, Japan). The fourth passage cells were used for the study.

### Characterization of isolated PDL cells as mesenchymal stem cells

The hPDLSCs of the fourth passage were detached and identified using specific surface antigen expression by flow cytometry. After the cells were harvested and transferred, they were fixed for 15 min in 4% paraformaldehyde. The cells were incubated with 3% bovine serum albumin and then with primary antibodies raised against CD105, CD90, CD73, CD34, CD45, and HLA-DR for 1 h, as described in our previous work [23]. The cells were washed with wash buffer, and the secondary antibody was added for 45 min at room temperature. Finally, the cells were washed three times and analyzed using a flow cytometer [24, 25].

### Multilineage differentiation

**Fig. 1:**
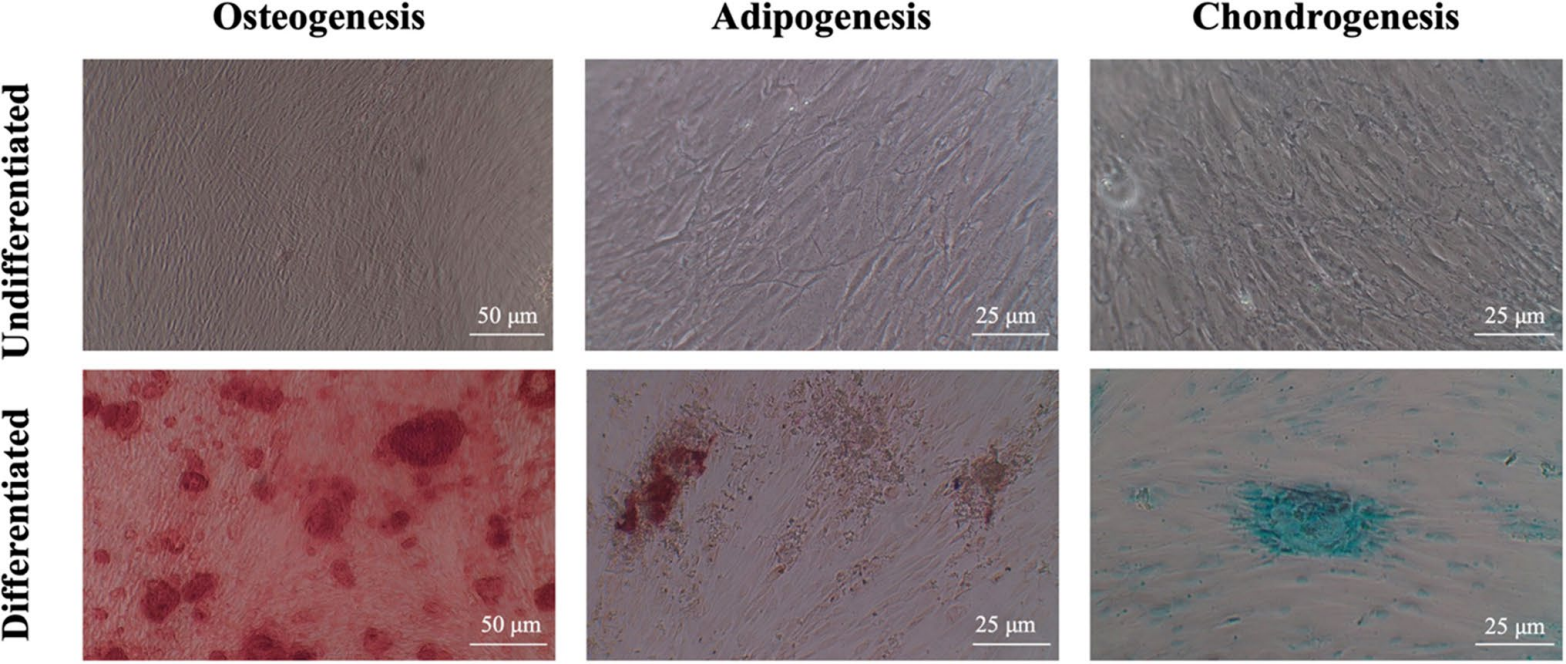
Inverted microscope images showing Alizarin red S (ARS) staining after osteogenic differentiation (100× original magnification with scale bar of 50 μm), Oil Red O staining after adipogenic differentiation and Alcian Blue staining after chondrogenic differentiation. (200× original magnification with scale bar of 25μm)

To analyze the in vitro multipotential differentiation ability of the characterized hPDLSCs, about 5 × 10^4^ cells/ml from the fourth passage cultures were cultured in 24-well plates using the commercially available kit (human mesenchymal stem cell functional identification kit, R&D Systems, Minneapolis, USA), media were changed every 3 days for 3 weeks to induce osteogenic, adipogenic, and chondrogenic differentiation, respectively. Cultures that were maintained in normal complete media served as reference. After 21 days, osteogenesis was demonstrated by mineralization and assessed by Alizarin red staining (Sigma-Aldrich), adipogenesis was evaluated with Oil Red O solution (Sigma-Aldrich) to detect accumulation of neutral lipid droplets, and chondrogenic differentiation was verified with Alcian blue staining (Sigma-Aldrich) to detect glycosaminoglycans [26]. All staining procedures were performed according to the manufacturer’s protocols. Wells were analyzed using inverted microscope (Axio Observer A.1, Zeiss, Oberkochen, Germany) and imaging was performed using a digital camera (Canon, Woodhatch, UK) (Fig. 1).

### Cell viability assay (MTT assay)

The cytotoxicity of the extracts to the hPDLSCs was assessed using the 3-(4, 5-dimethylthiazol-2-yl)-2, 5-diphenyltetrazolium bromide (MTT) assay. Briefly, 1 × 10^4^ hPDLSCs was added to 96-well plates with 180 μl of DMEM for 24 h. Then, extracts of the materials were added, and cells incubated at 37 °C in a 5% CO_2_ for 24, 48, or 72 h. The samples were incubated with 1 mg/ml of MTT for 4 h at the indicated time points. Then, 0.2 ml of dimethyl sulfoxide (DMSO) was added to each well to solubilize the formazan crystals obtained as a result of MTT reduction by the viable cells. The optical density (OD) value was measured by spectrophotometer at 570 nm [22]. The data obtained by each group was normalized based on cells + medium, and cell viability was calculated using the formula below [27].

$$=\frac{\textrm{OD}\ \textrm{values}\ \textrm{of}\ \textrm{experimental}\ \textrm{wells}\times 100}{\textrm{OD}\ \textrm{values}\ \textrm{of}\ \textrm{control}\ \textrm{wells}}$$

All samples were analyzed in triplicate, and the experiment was repeated three times.

### Cell migration/proliferation (wound healing assay)

In 6-well plates, 3× 10^5^ hPDLSCs were seeded in each well in complete culture medium. After reaching 100% confluence, a scratch was created in each well using a sterile pipette tip passing through the cell’s monolayer. The cells were washed with BPS to remove cell debris, and fresh medium was added to the control group. Material eluates were added at dilutions of 1:1, 1:2, and 1:4 to the cell’s monolayer in the study groups. The scratch in each well was imaged using inverted microscope; the images were captured at days 0, 1, 2, and 3 at the same place each time [23]. Wound area was calculated using image J software (National Institutes of Health, Bethesda, MD, USA), and percentage wound closure was calculated for each well [28]. Each group was evaluated in triplicate, and the data are presented as the mean of three separate experiments ± standard deviation (*SD*).

### Cell attachment/morphology (SEM)

hPDLSCs were seeded in 12-well plates at 2 × 10^5^ cell density on Biodentine, NeoPutty, and TheraCal PT discs for 72 h. Subsequently, discs were removed and carefully washed by BPS to remove unattached cells. hPDLSCs were then fixed using 4% glutaraldehyde for 2 h at 4 °C. Samples were finally dehydrated using increasing ethanol concentrations of 25, 50, 75, 95, and 100% for 5 min in each concentration. Samples were subjected to sputter-coating with gold using 15 mA for 4 min (HUMMER 8.0, Anatech, NV, USA). Discs without cells and those with cultured cells were analyzed by scanning electron microscopy (Leo Supra 55, Zeiss, Oberkochen, Germany) [29].

### Hydroxyl and calcium ion leaching evaluation

A 3-mm diameter polyethylene sterile catheter was sectioned horizontally at 2-mm intervals to give circular molds used for sealer disc preparation. Each mold was ligated with a non-waxed dental floss. Disc-shaped specimens (3-mm diameter × 2 mm thickness) were prepared by injecting the sealer inside the polyethylene mold (*n* = 5). Then, each disc was immersed individually in a sealed sterile glass test tube containing 5 ml deionized water (Water HPLC grade, Chemlab, Zedelgem, Belgium) and stored in an incubator (Titanox, Torre De’ Picenardi CR, Italy) at 37 °C. The leachates were collected from each tube on the 1st, 7th and 14th days for Ca^2+^ release and pH measurements. Specimens were then transferred to new tubes containing fresh deionized water [30, 31].

The pH-value of the leachate was evaluated for each material after each storage period via a pH meter (B 712 LAQUA twin compact pH meter, Horiba Scientific, Kyoto, Japan) that was initially standardized by buffered solutions (pH 7) and recalibrated before testing each new specimen. After specimen removal and shaking the storage tube for 5 s, 1 ml from each leachate was aspirated using a micropipette (TopPette, Dragon Laboratory Instruments, Beijing, China) and was placed in the pH meter measuring lens. For each sample, the pH was measured twice to calculate a mean value [30, 31].

Ca^2+^ release of the tested materials at the predetermined time intervals (*n* = 5) was measured using inductively coupled plasma-optical emission spectrometry (ICP-ms AGILENT 8800, Santa Clara, CA, USA) [23, 30, 31].

### Statistical analysis

All *in vitro* assays were performed in triplicates and analyzed in three independent experiments. After confirming the homogeneity of variance and normal distribution of the data, one-way ANOVA was performed followed by pair-wise Tukey’s post hoc test using Graph-Pad Prism v8.1.0 (GraphPad Software, San Diego, CA, USA). Data are expressed as mean ± standard deviations (*SD*). Statistical significance was considered at *p* < 0.05.

## Results

### Cell viability assay

**Fig. 2:**
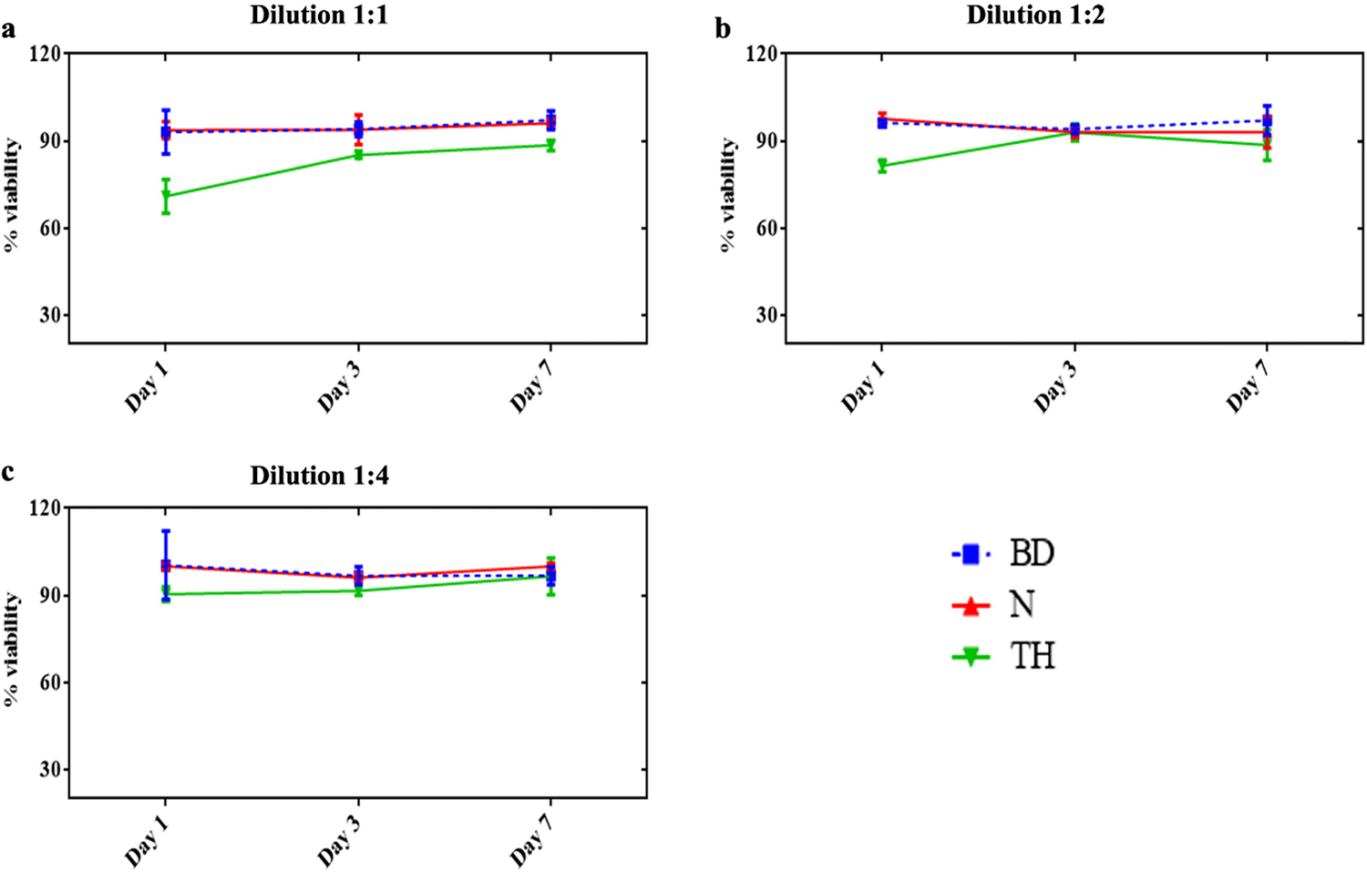
MTT assay showing the mean percentage of cell viability with standard deviation after exposure to the different repair materials. Data obtained by each group were normalized based on cells + medium (control). **a** Dilution 1:1. **b** Dilution 1:2. **c** Dilution 1:4. BD, Biodentine; N, NeoPutty; TH, TheraCal PT

For the 1:1 dilution significant difference regarding the percentages of cells of the three repair materials was obtained at day 1 (*p* < 0.0001), and days 3 and 7 (*p* <0.05). Biodentine and NeoPutty showed comparable results, which were significantly higher than for TheraCal PT at all observation periods (*p* < 0.005) except at day 7, where no significant difference (*p* > 0.05) between NeoPutty and TheraCal PT was found (Figure 2a). For dilutions of 1:2 and 1:4; Biodentine and NeoPutty showed significantly higher cell viability than TheraCal PT at day 1 (*p* < 0.05), while at days 3 and 7 all the three repair materials revealed comparable results (*p* > 0.05; Fig. 2b, c).

### Cell migration assay

**Fig. 3:**
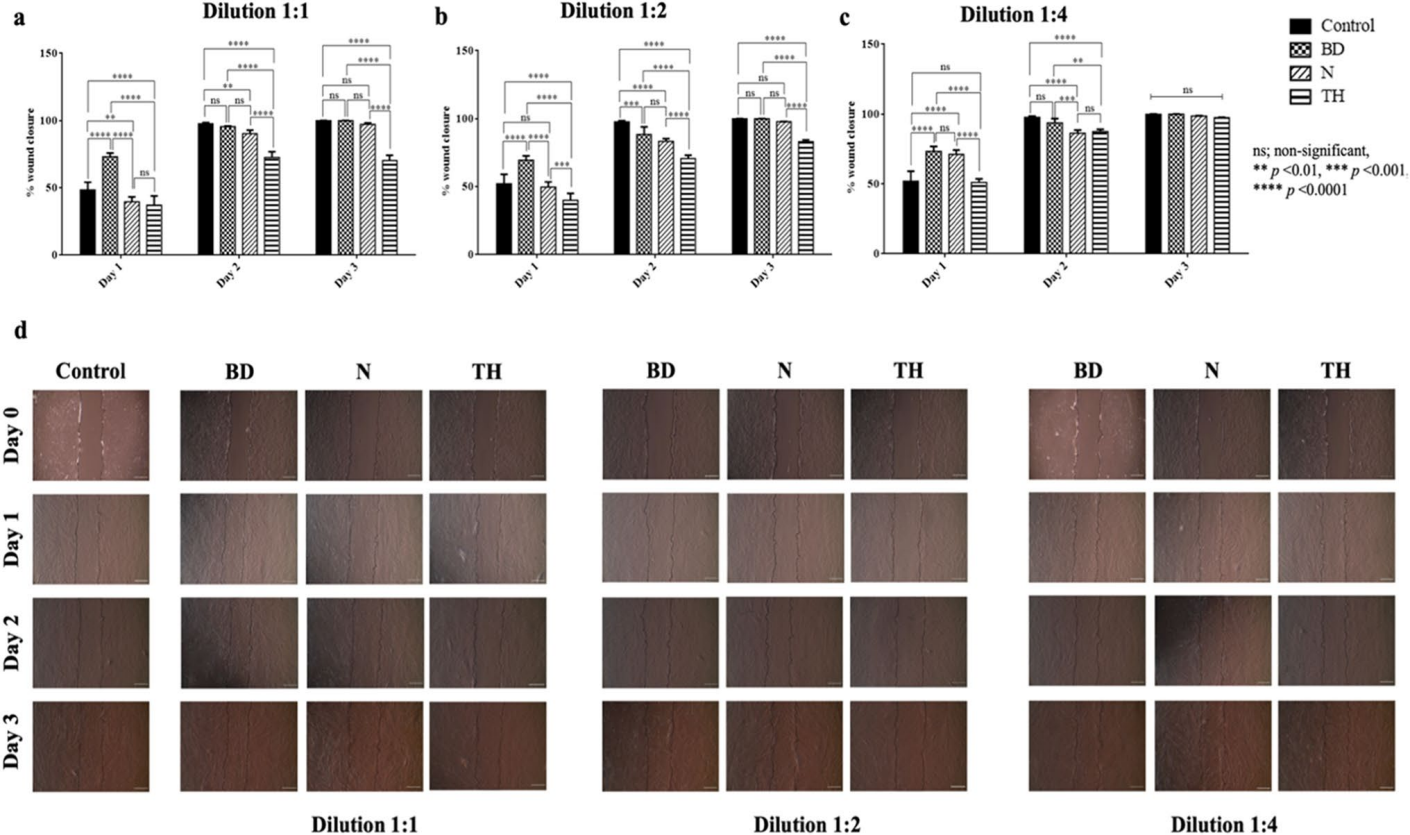
Bar graphs showing the percentage of wound closure indicating the migration of stem cells to the scratched area on days 1, 2, and 3. **a** Dilution 1:1. **b** Dilution 1:2. **c** Dilution 1:4. **d** Images of the scratch healing of all groups acquired at days 1, 2, and 3 with a phase-contrast microscope (TCM 400) at magnification of 40×. Cell migration area was calculated using Image J software, and wound closure percentage was calculated for each well. BD, Biodentine; N, NeoPutty; TH, TheraCal PT

The results of the cell migration assay (Fig. 3) revealed that for the dilution 1:1 at day 1, Biodentine showed the highest percentage of wound closure compared to the control group and the other two repair materials (*p* < 0.0001). At days 2 and 3, Biodentine and NeoPutty showed comparable results, while TheraCal PT showed the lowest migration of stem cells into the scratched area (*p* < 0.0001; Fig. 3a). For the dilution 1:2, the same trend as for dilution 1:1 was obvious, as Biodentine showed a higher percentage of stem cell migration than the control group and the other repair materials (*p* < 0.0001). At day 2 all repair materials showed lower percentages of wound closure than the control group. TheraCal PT displayed the lowest percentage of open wound closure (*p* < 0.0001). At day 3, Biodentine and NeoPutty showed comparable results to the control group, while TheraCal PT displayed the lowest values of stem cell migration (*p* < 0.0001; Fig. 3b). For the dilution 1:4, at day 1, Biodentine and NeoPutty showed higher percentage of cell migration than the control and TheraCal PT (*p* < 0.0001). At day 2, Biodentine showed the highest percentage of stem cell migration, which was comparable to the control group (*p* < 0.0001). No significant difference was found between all groups at day 3 (*p* > 0.05; Fig. 3c).

### Cell attachment/morphology

**Fig. 4:**
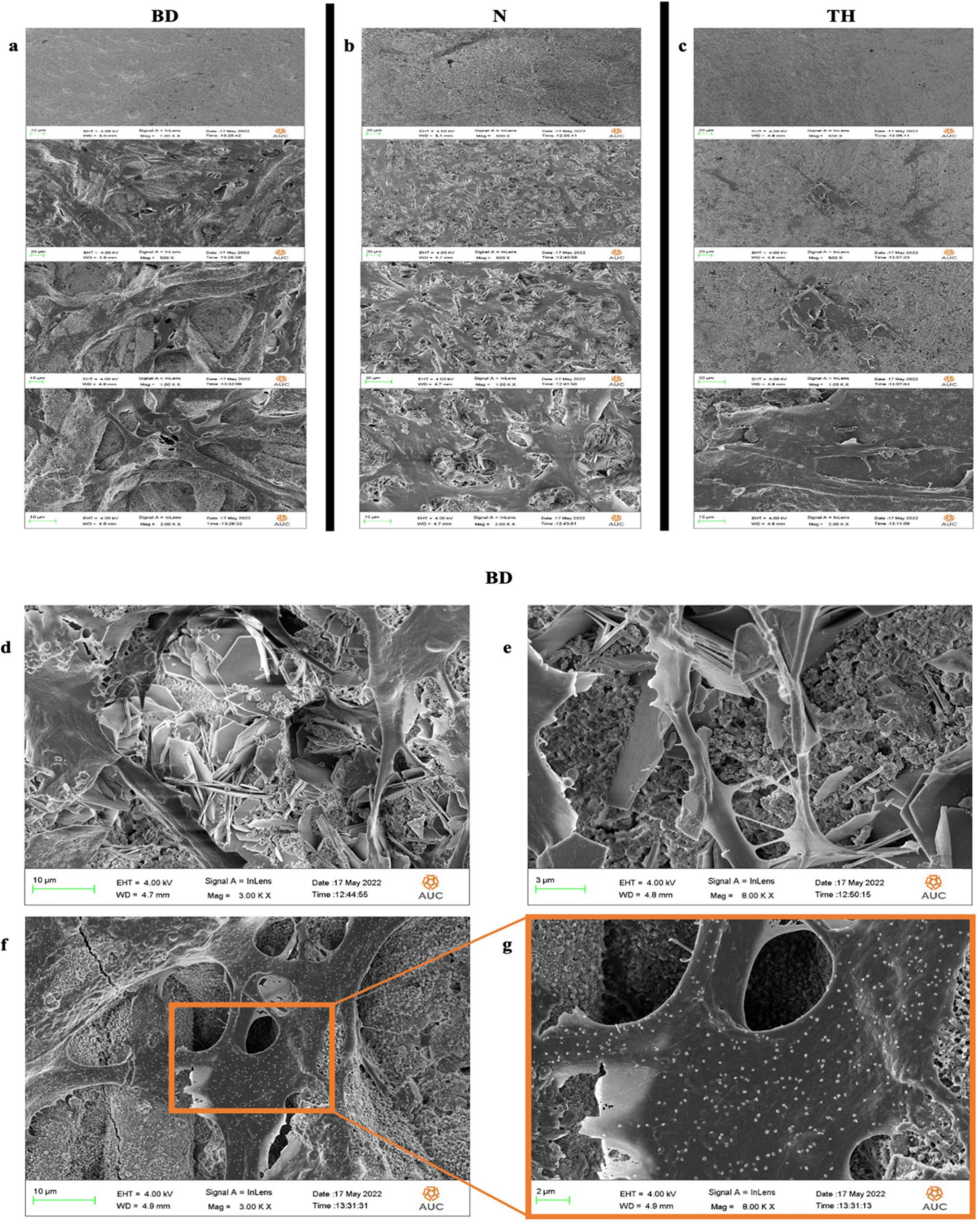
Scanning electron microscopy images showing the attachment and the morphology of hPDLSC (magnification range 500×–2.00K×). **a** BD, Biodentine. **b** N, NeoPutty. **c** TH, and TheraCal PT **d**. Calcium hydroxide crystals in the Biodentine group (magnification 3.00K×). **e** Evident cell spreading and intercellular communications in the Biodentine group (magnification 8.00K×). (f) Mineralized nodule deposition by hPDLSCs was evident in Biodentine group (magnification 3.00K×). (g) Magnified image of the calcific nodules’ deposition (magnification 8.00K×)

hPDLSCs attached favorably on Biodentine (Fig. 4a, d and e) and NeoPutty (Fig. 4b). Cell spreading, intercellular communications, and pseudopodia formation were evident on both materials (Fig. 4e, f). Contrarywise, poor cell attachment of round-shaped cells with poorly branched processes was evident on TheraCal PT (Fig. 4c). Mineralized nodule deposition by hPDLSCs was evident in Biodentine group (Fig. 4f, g).

### pH values

**Fig. 5:**
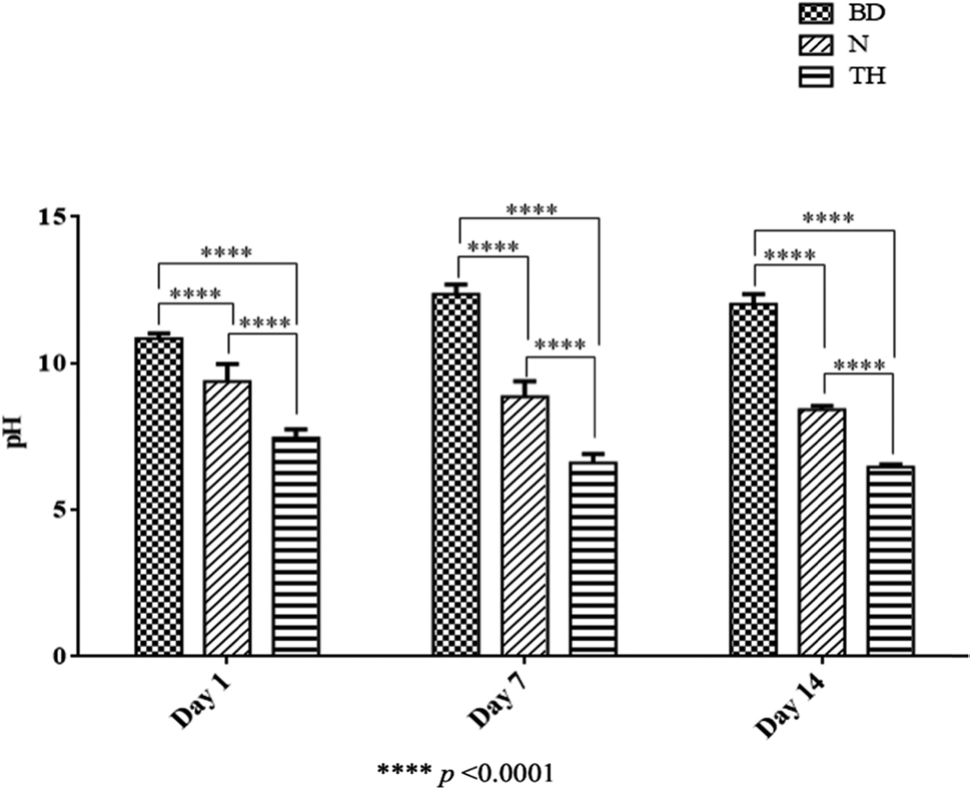
Bar graph showing the pH values of the three repair materials given as means and standard deviations at days 1, 7, and 14. BD, Biodentine; N, NeoPutty; TH, TheraCal PT

At all observation times, Biodentine obtained the significantly highest pH values of all repair materials (*p* < 0.0001; Fig. 5). pH values of NeoPutty were significantly higher than those of TheraCal PT (*p* < 0.0001; Fig. 5).

### Calcium ion release

**Fig. 6:**
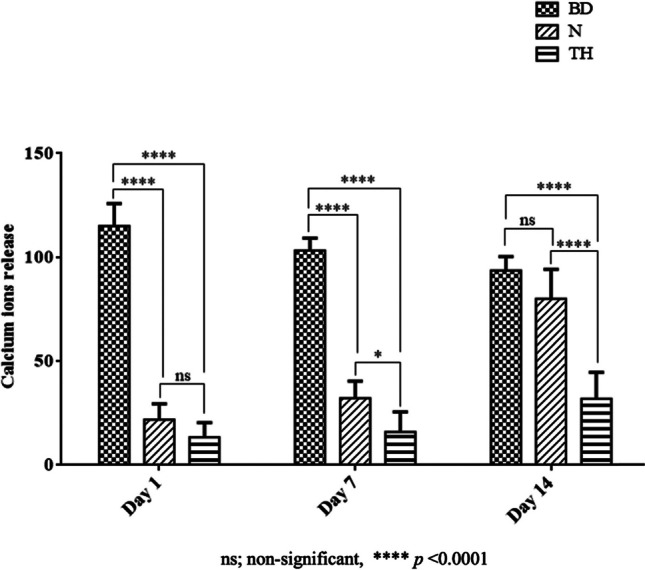
Bar graph showing the calcium ion release values of the repair materials given as means and standard deviations at days 1, 7, and 14. BD, Biodentine; N, NeoPutty; TH, TheraCal PT

At all observation times, calcium ion release of Biodentine was significantly higher than of the other two materials (*p* < 0.0001; Fig. 6). NeoPutty and TheraCal PT showed comparable results at day 1 (*p* > 0.05), while at days 7 and 14, NeoPutty displayed higher calcium ion release than TheraCal PT (*p* < 0.0001; Fig. 6)

## Discussion

The biological properties and bioactive potential of hydraulic calcium silicate–based materials have been documented by numerous studies. Still, the rapid introduction of new material compositions with different setting mechanisms into the market calls for an updated biological and chemical profiling of each new composition before its clinical use. This highlights the importance and relevance of *in vitro* studies as a preliminary approach for the assessment of new materials. However, results from these assays should be interpreted with caution, since the behavior of the tested materials could be potentially influenced by a number of external factors in the clinical setting [23].

### Experimental setup

The present study investigated the effects of three calcium-silicate based materials on the viability, migration, proliferation, morphology, and adhesion of hPDLSCs. Although recommendations laid down in the ISO specification 10993-5 suggest using established cell lines in cytotoxicity assays, freshly isolated human hPDLSCs were used in this study as they may get in contact with such materials *in vivo* [23]. Although present in a lesser quantity than periodontal ligament fibroblasts and osteoblasts, hPDLSCs are considered a prime cell source for periodontal regeneration [32] and are supposed to be involved in healing of apical periodontitis and tissue regeneration [33]. This approach is in line with several previous comparable studies [20, 21, 34], while one study used human bone marrow-derived mesenchymal stem cells instead of hPDLSCs [35]. Dental stem cells, as a subpopulation of multipotent mesenchymal stromal cells (MSCs), share a specific mesenchymal-like phenotype, but also exhibit individual characteristics, which could result in a different response to external stimuli [36]. For example, hPDLSCs exhibit a higher osteogenic potential compared to hDPSCs [19] and multipotent stem cells derived from pulp tissue of human exfoliated deciduous teeth (SHEDs) [37]. This justifies the need for a separate assessment and categorization of the biological behavior of the different dental stem cell variants.

The minimum criteria for the identification of MSCs have been developed by the International Cellular Therapy Association. Accordingly, MSCs should adhere to plastic surfaces under standard culture conditions and express CD105, CD73, and CD90, no expression of CD45, CD34, CD14, and CD11b. MSCs should be able to differentiate into different cells such as osteoblasts, adipocytes, and chondroblasts in vitro [38]. Therefore, primary antibodies raised against CD105, CD90, CD73, CD34, and CD45 were used in the present study for characterization of the isolated PDL cells as mesenchymal stem cells. This approach has been further described in more detail previously [23].

According to ISO standards, cell viability tests can be evaluated after 24, 48, and 72 h. Although the cell viability test periods are generally stated as the 1st and 3rd days in the literature, the cell viability was evaluated at the end of the 7th day in this study in order to evaluate the long-term toxic effects of the materials [39]. Other comparable studies also investigated cell viability after 7 days [34, 35]. The use of three dilutions (1:1, 1:2, and 1:4) was performed to simulate the clinical conditions, in which the tested materials can be placed on the remaining dentin thicknesses of 0.01 to 0.25 mm or directly on pulp exposures. Therefore, the concentration of the material that reaches viable pulp tissue may differ [17].

It is well known that bioactive materials release substances that could potentially delay or enhance the healing process in the periradicular tissues. For this reason, the wound healing assay was performed to predict how the coordinated migration of hPDLSCs would occur after exposure to the tested materials. There is a direct correlation between cell migration and cell viability [40].

All tested materials in the present study were incubated as set discs. According to a recent systematic review, this is in accordance with most *in vitro* studies on the biological interaction between CSBMs and dental stem cells [41]. Although the use of freshly mixed materials can better predict the biologic response of dental stem cells, the use of set materials is superior for prediction of their delayed/long-term response [42]. Future studies should include both preparations, to provide a comprehensive biological profiling of the tested repair materials [43].

### Findings

Regarding cell migration for the 1:1 and 1:2 dilutions at day 1, pH value, and calcium ion release, Biodentine achieved significantly better results than both other materials. With regard to cell viability of the 1:1 and 1:2 dilutions and cell attachment, Biodentine and NeoPutty were significantly superior compared to TheraCal PT. Therefore, the null hypothesis of this study was rejected.

The results obtained for Biodentine are corroborated by several previous studies, which confirmed the excellent level of cytocompatibility of this CSBM [20, 35, 44–46]. Biodentine possesses the potential to induce proliferation and osteogenic differentiation of dental stem cells [35, 46], as this CSBM has been shown to upregulate the odontogenic marker dentin sialophosphoprotein [20, 45]. A consistent finding is that Biodentine promoted mineralized nodule deposition in about 21 days [20, 44] and that exposure of Biodentine to phosphate-buffered solutions resulted in precipitation of apatite crystalline structures on its surface [47, 48]. Also, the good attachment of stem cells on the surface of Biodentine, as found in the present study, is in agreement with previous reports [35].

To a certain extent, these properties of Biodentine are related to the distinct calcium ion release and the relatively high pH of this CSBM, as Ca^2+^ is necessary for the differentiation, proliferation, and mineralization of cells [34]. Ca^2+^ has an impact on the activity of pyrophosphatase, which induces dentin mineralization [49]. An alkaline pH has an impact on transforming growth factor-β1 (TGF-β1) release, and TGF-β1 may induce osteoblastic proliferation and differentiation of odontoblasts [50]. The present results reveal that both pH values as well as calcium ion release were significantly higher for Biodentine than for the two other CSBMs tested. These findings are corroborated by previous studies, confirming the capacity of Biodentine to release calcium ions [31, 46, 47, 51, 52] and to create an alkaline pH [31, 44, 47, 50, 52, 53].

NeoPutty obtained similar results compared to Biodentine regarding cell viability, cell migration for dilutions of 1:1 and 1:2 after day 1 and for the 1:4 dilution at all observation times, and cell attachment. To date, limited scientific evidence regarding the biological properties of NeoPutty is available, Sun et al. [18] reported that NeoPutty exhibited a higher biocompatibility than another CSBMs (EndoSequence BC RRM putty (Brasseler, Savannah, GA, USA), while Lozano-Guillén et al. [17] reported similar biocompatibility of NeoPutty to its predecessor (NeoMTA Plus) and MTA, both studies used human dental pulp stem cells [54]. In line with these findings, another study investigated the effects of NeoPutty on human dental pulp stem cells and human periodontal ligament fibroblasts and found that these repair materials showed acceptable cytocompatibility profiles on both cell types [18]. It is worthy to mention that NeoPutty contains calcium aluminate that has been shown to have adequate biocompatibility after subcutaneous implantation in rats [55] and also supported the acquisition of osteogenic cell phenotypes in vitro [56].

According to the present results, the resin-modified material TheraCal PT showed significantly inferior results than Biodentine in all tests and displayed significantly worser results than NeoPutty regarding cell migration for the 1:4 dilution and the 1:1 and 1:2 dilutions after day 1, also for the cell attachment and pH value results. These observations are in full congruence with previous studies [31, 34] but partially disagrees the results of two other investigations [20, 21] that demonstrated favorable cytocompatibility on human DPSCs with mineralized nodule formation and bioactive properties [20, 21], as well as upregulation of the osteogenic markers osteonectin and runt-related transcription factor 2 [20]. Contrarily, Küden et al. [34] showed that TheraCal PT decreased the number of viable hDPSCs significantly from day 1 to day 3, and at day 7 viable cells were no longer detected. The authors correlated this adverse effect to the time-dependent monomer release of TheraCal PT and showed that this material released marked amounts of TEGDMA (triethylene glycol dimethacrylate) at days 3 and 7 [34]. Moreover, in accordance with the present results, Elbanna et al. [31] reported that TheraCal PT merely slightly increased the pH value up to about 8.7 after 14 days and that the calcium ion release was very limited, as also reported by Küden et al. [34]. On the whole, the authors concluded that TheraCal PT exhibited only limited bioactivity [31].

In general, the present findings support the results of a systematic review [7] in as far as the two resin-free CSBMs tested consistently provided significantly better results than the resin-based material.

#### Conclusions

The two resin-free CSBMs used in this study are biocompatible with the potential to induce proliferation and adhesion of hPDLSCs, as opposed to the resin-based material TheraCal PT. Therefore, Biodentine and NeoPutty show potential for use as an endodontic repair material.

## Data Availability

Data available on request from the authors.
